# Investigating the Link between Alpha-1 Antitrypsin and Human Neutrophil Elastase in Bronchoalveolar Lavage Fluid of COVID-19 Patients

**DOI:** 10.3390/cimb44050143

**Published:** 2022-05-10

**Authors:** Maura D’Amato, Valentina Vertui, Laura Pandolfi, Sara Bozzini, Tommaso Fossali, Riccardo Colombo, Anna Aliberti, Marco Fumagalli, Paolo Iadarola, Camilla Didò, Simona Viglio, Federica Meloni

**Affiliations:** 1 Department of Molecular Medicine, University of Pavia, 27100 Pavia, Italy; maura.damato90@gmail.com; 2 Department of Internal Medicine and Medical Therapeutics, University of Pavia, 27100 Pavia, Italy; valentina.vertui@gmail.com (V.V.); pandolfi.la@gmail.com (L.P.); didocamilla@icloud.it (C.D.); f.meloni@smatteo.pv.it (F.M.); 3 Laboratory of Respiratory Disease, Cell Biology Section, Fondazione IRCCS Policlinico San Matteo, 27100 Pavia, Italy; s.bozzini@smatteo.pv.it; 4 Department of Anesthesiology and Intensive Care, ASST Fatebenefratelli Sacco, Luigi Sacco Hospital, University of Milan, 20121 Milan, Italy; tommaso.fossali@asst-fbf-sacco.it (T.F.); riccardo.colombo@asst-fbf-sacco.it (R.C.); 5 Division of Anesthesiology and Intensive Care 1, Fondazione IRCCS Policlinico San Matteo, 27100 Pavia, Italy; an.aliberti@smatteo.pv.it; 6 Department of Biology and Biotechnologies “L. Spallanzani”, University of Pavia, 27100 Pavia, Italy; marco.fumagalli@unipv.it (M.F.); piadarol@unipv.it (P.I.); 7 Transplant Unit, Fondazione IRCCS Policlinico San Matteo, 27100 Pavia, Italy

**Keywords:** lung, alpha-1-antitrypsin (AAT), human neutrophil elastase (HNE), COVID-19, broncho-alveolar lavage (BAL), Neutrophils Extracellular Traps (NETs), Liquid Chromatography Mass Spectrometry (LC-MS), neutrophils, histones

## Abstract

Neutrophils play a pathogenic role in COVID-19 by releasing Neutrophils Extracellular Traps (NETs) or human neutrophil elastase (HNE). Given that HNE is inhibited by α1-antitrypsin (AAT), we aimed to assess the content of HNE, α1-antitrypsin (AAT) and HNE–AAT complexes (the AAT/HNE balance) in 33 bronchoalveolar lavage fluid (BALf) samples from COVID-19 patients. These samples were submitted for Gel-Electrophoresis, Western Blot and ELISA, and proteins (bound to AAT or HNE) were identified by Liquid Chromatography-Mass Spectrometry. NETs’ release was analyzed by confocal microscopy. Both HNE and AAT were clearly detectable in BALf at high levels. Contrary to what was previously observed in other settings, the formation of HNE–AAT complex was not detected in COVID-19. Rather, HNE was found to be bound to acute phase proteins, histones and C3. Due to the relevant role of NETs, we assessed the ability of free AAT to bind to histones. While confirming this binding, AAT was not able to inhibit NET formation. In conclusion, despite the finding of a high burden of free and bound HNE, the lack of the HNE–AAT inhibitory complex in COVID-19 BALf demonstrates that AAT is not able to block HNE activity. Furthermore, while binding to histones, AAT does not prevent NET formation nor their noxious activity.

## 1. Introduction

SARS-CoV-2 is a new type of Coronavirus responsible for the COVID-19 pandemic. It can easily spread to the lower respiratory system causing a range of clinical manifestations that vary from paucisymptomatic pneumonia to acute respiratory distress syndrome (ARDS) [1]. SARS-CoV-2 is also related to systemic involvement, targeting kidneys, heart, and the central nervous system, as well as promoting thrombosis [2]. COVID-19 is characterized by a strong innate response that leads to a cytokine storm with massive recruitment of inflammatory cells [3]. Specifically, high levels of IL-6 and IL-8 have been found in bronchoalveolar lavage (BAL) and in plasma from SARS-CoV-2 infected patients [4,5]. These cytokines are pro-inflammatory and can act as chemoattractants for neutrophils, which are abundant in alveoli and in BAL in patients with severe COVID-19 [4,6]. Neutrophils are the first innate cells to run against foreign pathogens [7], and they are one of the major causes of endothelial and epithelial damage in SARS-CoV-2 infection. These cells exert their protective activity by phagocytosis and externalization of antimicrobial enzymes including neutrophil elastase (HNE), cathepsin G (Cat G), myeloperoxidase (MPO), and reactive oxygen species (ROS) [8]. Neutrophil granules’ enzymes, especially HNE and ROS, are responsible for direct tissue damage in many lung diseases [9,10,11,12]. A role for HNE in COVID-19 pathogenesis has been proposed but data are still limited [13]. Increased levels of HNE have been detected in nasopharyngeal swabs and in the peripheral blood of infected patients [14,15]. Furthermore, HNE may be involved in SARS-CoV-2 virulence as protease cleavage of the Spike protein is required for SARS-CoV-2 to enter host cells and the virus mutation 614G creates a new neutrophil elastase cleavage site in the Spike protein that can increase virus spread [16]. In healthy individuals, HNE is balanced (in a 1:1 stoichiometric ratio) by α1-antitrypsin (AAT), a specific inhibitor that covalently binds to HNE to form a stable complex (HNE–AAT) [12]. Previous evidence showed that the overexpression of HNE in some lung diseases creates an imbalance in its ratio with AAT, contributing to lung infection and tissue destruction [12]. A potential protective role of AAT in COVID-19 was first hypothesized by Vianello and Braccioni [17]. Specifically, AAT could prevent the entry of SARS-CoV2 mediated by host transmembrane protease serine-type 2 (TMPRSS2) [18,19]. Furthermore, AAT has a known anti-inflammatory role, and highly sialylated M0 and M1 AAT glycosylated forms were found in plasma from SARS-CoV-2 positive patients. Moreover, in COVID-19, an imbalance between cytokines, specifically IL6 and AAT at the plasmatic level, has been associated with severe disease [20]. The latest neutrophil weapon discovered is NETosis, a form of programmed death that aims to create neutrophilic extracellular traps (NETs) [21]; although recently a viable form of NETosis has been described [22]. NETs are antimicrobial structures formed by a chromatin scaffold “decorated” with proteins and enzymes, which are usually stored in neutrophils’ cytoplasmatic granules [21]. NETs can be stimulated in vivo by foreign pathogens, ROS, lipopolysaccharide (LPS), cytokines, chemokines, and in vitro by phorbol myristate acetate (PMA), calcium, and potassium ionophores [23]. Both neutrophilia (expressed as augmented neutrophils count or elevated neutrophil/lymphocyte ratio) [24,25] and NETs’ biomarkers relate to severe COVID-19 [26,27]. In fact, dysregulation, and overproduction of NETs lead to tissue damage on both the epithelial and endothelial sides [28]. Specifically, in COVID-19 NETs play a crucial role in direct lung damage, but they also seem to be responsible for immune-thrombosis and multi-organ involvement [29,30]. The importance of NETosis in COVID-19 is testified by the presence of NETs in BAL [26], patients’ sera [27], and post-mortem lung specimens [31]. In addition, incubation of SARS-CoV-2 and sera from infected patients with human neutrophils stimulates NETs, suggesting that SARS-CoV-2 induces a pro-NETotic milieu [27,32]. Aracnjo and colleagues [32] showed that SARS-CoV-2 can induce the release of a vast amount of ROS from neutrophils, which contributes to tissue damage. However, ROS are also known to stimulate NETs, creating a vicious cycle maintaining NETosis and inflammation. In support of this, the impairment of oxidant/antioxidant balance in COVID-19 was found to be related to inflammatory markers and worse outcome in COVID-19 [33].

The exact role of AAT in the direct protection from tissue damage induced by NETs is still poorly defined. Data on the ability to inhibit the process of NETosis are controversial: some studies on AAT-deficient animals suggests that AAT might inhibit NET formation, while others, obtained on human in vitro stimulated neutrophils, show no significant effect [34,35]. On the other hand, a recent study demonstrated that cleavage fragments of AAT, which are generated in human placenta by high-temperature requirement serine protease A1, can inhibit NET generation by mature human neutrophils [36] as well as another natural anti-protease factor, secretory leukocyte protease inhibitor (SLPI) [37]. Even if AAT is not able to inhibit NET formation, it has been shown to modulate their size and adhesion [34] while there are conflicting data on the ability of AAT to counteract NET- associated HNE activity. When bound to NETs, HNE is able to degrade plasminogen, reduce plasmin formation, and decrease fibrinolysis [38]. Other evidence obtained on bronchoalveolar lavage samples from Cystic Fibrosis patients suggest that NET-associated HNE proteolysis is significantly increased after treatment with DNAse, suggesting that NET-DNA binds and inhibit HNE [39]. AAT has been shown to bind and successfully inhibit NET-associated elastase in vitro [40], but its activity in the context of COVID-19 pneumonia has yet to be clarified.

The aim of the present work was to investigate the relationship between the major protease (HNE) and AAT in patients affected by severe COVID-19 requiring mechanical ventilation in an intensive care department. To this purpose, BALf samples from 33 COVID-19 patients were compared with those obtained from patients with other neutrophilic small airway inflammation, i.e., Bronchiolitis obliterans syndrome (BOS) (*n* = 10). The levels of HNE, AAT, HNE–AAT complex, and NETs were assessed, and all proteins found bound to HNE were identified. Finally, the real capacity of AAT to inhibit HNE in these patients and its potential to modulate or bind NETs were analyzed.

## 2. Materials and Methods

### 2.1. Reagents

Antibodies for detection of AAT, HNE, and secondary anti-mouse antibodies were obtained from Abcam (Cambridge, UK) and Thermo Scientific (Rockford, IL, USA). Bicinchoninic acid (BCA) protein assay kit was obtained from Thermo Scientific (Rockford, IL, USA). Unless otherwise stated, all other analytical grade reagents were purchased from Sigma-Aldrich (St. Louis, MO, USA). Double-distilled water used for the preparation of all buffers was prepared with a Millipore (Bedford, MA, USA) Milli-Q purification system.

### 2.2. Patients

For this study, 33 BALf samples from hospitalized patients (mean age 59.66 ± 20; Table 1), collected from March 2020 to December 2021, were evaluated. The patients were admitted with severe pneumonia to the intensive care units at ASST Fatebenefratelli Sacco, Milan or Foundation IRCCS San Matteo, Pavia with positive SARS-CoV-2 polymerase chain reaction (PCR) tests. The sample collection was approved by the Local Ethics Committee (Comitato Etico di Area 1prot. 20100005334 as for Sacco and 20200046007 as for IRCCS San Matteo) and was in accordance with the ethical principles in the Helsinki declaration. Informed consent was collected from all participants or next of kin. Demographic and clinical data were collected retrospectively from the patients’ charts.

BALf was collected from ICU patients at admission, usually before starting experimental treatment strategies. BALf was treated according to guidelines during the first and second wave of COVID-19, with approved therapies as steroids and low molecular weight heparin, and during third wave, with tocilizumab. All procedures for the collection, transport, and preparation of the samples were carried out according to the restrictions and protocols required by the Italian law.

The demographic and clinical features (including age, gender, % of macrophages, lymphocytes, neutrophils, eosinophils, days after incubation and P/F) of patients considered in this study are detailed in Table 1.

### 2.3. BALf Collection and Processing

BALf collection was performed as previously described [41]. Briefly, the distal tip of the bronchoscope was wedged into the middle lobe or lingular bronchus, and a total of 150 mL of warm sterile saline solution was instilled in five subsequent 30 mL aliquots which were sequentially retrieved by gentle aspiration. The first aliquot collected (20 mL) was used for a series of analyses which included microscopic and cultural examination of common bacteria and fungi and direct/cultural investigations of respiratory viruses. The returned fluid from the second to the fifth aliquots was pooled and further processed as BALf. Cells were recovered by centrifugation at 1500 rpm for 10 min and supernatants were divided in aliquots (30 mL each) which were stored at −80 °C immediately after processing, until use.

### 2.4. Neutrophil Isolation and NETosis

Neutrophils were isolated from the peripheral blood of healthy donors following the protocol already published [26]. To induce NETosis, 2.5 × 10^6^ neutrophils were cultivated with the addition of 100 nM PMA (Sigma-Aldrich, St.Louis, MO, USA) with or without AAT (500 µg/mL) for 4 h at 37 °C. 

### 2.5. BCA Protein Assay

The exact protein concentration in each sample was determined by applying the bicinchoninic acid (BCA) assay [42] using a calibration curve produced by bovine serum albumin (BSA), in the range of concentration between 5 and 25 µg /mL.

### 2.6. 1D-PAGE

An aliquot of each sample (20 µg of protein) was submitted to protein precipitation with trichloroacetic acid (TCA), according to Yvon et al. [43]. After centrifugation, the pellet was reconstituted in 10 µL of 50 mM TrisHCl pH 8.3 containing 5% 2-mercaptoethanol, 2% sodium dodecyl sulphate (SDS), 0.1% bromophenol blue (BPB) and 10% glycerol.

Samples were incubated at 95 °C for 5 min and then loaded on gel slabs. Electrophoresis was performed according to Laemmli [44] in 5% stacking gel and 12.5% running gel by applying a voltage of 150 V for 1 h. Gels were stained with colloidal Coomassie G-250, according to Candiano et al. [45].

### 2.7. Western Blotting

Ten micrograms of proteins were precipitated by the addition of 1.22 M TCA and the pellet recovered after centrifugation was submitted to SDS-PAGE. Protein bands were transferred into a Millipore polyvinyl divinyl fluoride (PVDF) membrane (Billerica, MA, USA) by using a trans blot turbo system (BioRad Laboratories, Segrate (MI), Italy). After 1 h incubation in 5% milk diluted in phosphate buffer saline (PBS) and three washes with phosphate buffer saline containing 0.1% Tween 20 (PBST), the membrane was incubated overnight with AAT antibody (ab9400; Abcam, Cambridge, UK) at a 1:2500 dilution in 1% milk. The membrane was washed three times with PBST (10 mL), incubated with the secondary antibody, rabbit anti-mouse IgG H&L (HRP) (ab6728, Abcam), at a 1:5000 dilution in 1% milk in PBST, for 1 h at room temperature. The membrane was washed again (three times) with PBS and incubated in ECL Westar ηC Ultra (Cyanagen, Bologna, Italy) solution according to the provided protocol. The same procedure was applied for the identification of free and complexed HNE by using the anti HNE antibody (MA5-2548, Invitrogen, Waltham, MA, USA) at a 1:1000 dilution and secondary antibody anti-rabbit (ab6721, Abcam) at a 1:3000 dilution. All immunoblots were acquired with the Image Quant LAS 4000 analyzer (GE Healthcare, Chicago, IL, USA).

### 2.8. In-Situ Digestion

Enzymatic digestion of proteins was performed as previously described [46]. Briefly, the selected bands were carefully excised from the gel, placed into Eppendorf tubes, broken into small pieces, and washed with 100 mM ammonium bicarbonate (AmBic) buffer pH 7.8 containing 50% acetonitrile (ACN) until complete de-staining was achieved. The gel pieces were then dehydrated by adding 200 µL of ACN until they became opaque-white color. Acetonitrile was finally removed, and gel pieces were dried under vacuum for 10 min and then rehydrated by adding 75 µL of 100 mM AmBic buffer pH 7.8, containing 20 ng/µL sequencing grade trypsin (Promega, Madison, WI, USA). The digestion was performed overnight upon incubation of the mixture at 37 °C and the resultant peptides were extracted from the gel matrix by a three-step sequential treatment with 50 µL of 50% ACN, 5% trifluoroacetic acid (TFA) in water, and finally with 100% ACN. Each extraction involved 10 min of stirring followed by centrifugation and removal of the supernatant. All supernatants were pooled, dried, and stored at −80 °C until mass spectrometric analysis. At the moment of use, the peptide mixture was solubilized in 0.1% formic acid (FA).

### 2.9. Liquid Chromatography Tandem Mass Spectrometry (LC-MS/MS)

Analyses were performed on a liquid chromatography-mass spectrometry system (Thermo Finnigan, San Jose, CA, USA) consisting of a thermostated column, a surveyor autosampler controlled at 25 °C, a quaternary gradient surveyor MS pump equipped with a diode array (DA) detector, and a linear trap quadrupole (LTQ) mass spectrometer with electrospray ionization (ESI) ion source controlled by Xcalibur software 1.4 (Thermo Fisher Scientific, Waltham, MA, USA). Analytes were separated by reverse-phase high performance liquid chromatography (RP-HPLC) on a Jupiter (Phenomenex, Torrance, CA, USA) C18 column (150 × 2 mm, 4 µm, 90 Å particle size) using a linear gradient (2–60% solvent B in 60 min) in which solvent A consisted of 0,1% aqueous FA and solvent B of ACN containing 0.1% FA. The flow rate was 0.2 mL/min. Mass spectra were generated in positive ion mode under constant instrumental conditions: source voltage 5.0 kV, capillary voltage 46 v, sheath gas flow 40 (arbitrary units), auxiliary gas flow 10 (arbitrary units), sweep gas flow 1 (arbitrary units), capillary temperature 200 °C, tube lens voltage −105 V. MS/MS spectra, obtained by collision-induced dissociation (CID) studies in the linear ion trap, were performed with an isolation width of 3 Th *m*/*z*, the activation amplitude was 35% of ejection RF amplitude that corresponds to 1.58 V. Data processing was performed using Peaks studio 4.5 software.

### 2.10. Preparation of Fluorescent Alpha 1-Antitrypsin

Protein was fluoresceinated following the protocol suggested by the company (Merck Millipore, Burlington, MA, USA, protocol 124546). In brief, 1 mg of AAT was reconstituted in 50 µL of PBS (20 µg/µL). 1 mg of powder FITC was dissolved in 2 mL of 0.1 M Na_2_HPO_4_ and incubated in a water bath at 25 °C. 30 µg of AAT were added to 7.5 µL of 0.2 M Na_2_HPO_4_ and 15 µL of FITC. The solution was brought to pH 9.5 by adding 3 µL of 0.1 M Na_3_PO_4_. Finally, 5.5 µL of 0.145 M NaCl was added. The solution was placed in a water bath and protected from light for 30 min and subsequently cooled on ice. Finally, the protein was filtered on Microcon centrifugal filters. 

### 2.11. Measurement of Elastase Activity Using a Nanodrop System

The elastase activity assay was carried out on all BALf samples that were analyzed by using a NanoDrop system (CLARIOstar, Thermo Fisher Scientific, Waltham, MA, USA). 20 µL of BALf was lyophilized on a Speedvac and the pellet was resuspended by adding 49.5 µL of incubation buffer (50 mM Tris pH 7.8; 500 mM NaCl) and 0.5 µL of MeOSuc-Ala-Ala-Pro-Val-pNA (Thermofisher Scientific, Waltham, MA, USA) 200 mM substrate solution. The samples were incubated for 30 min at room temperature and the reaction was stopped by adding 5 µL of TCA. Samples (5 µL) were read at 410 nm using the incubation buffer as blank. The activity units were calculated by reference to a calibration curve prepared reading at the same wavelength scalar amounts (in the range from 0.001 to 0.025 mM) of p-NA. One unit corresponds to the number of µmoles of p-NA released per min.

### 2.12. ELISA Assays 

To quantify HNE, the Human PMN Elastase ELISA Kit (Abcam, ab119553) was used following the instructions of the company. The ability of AAT to bind to hypothetical NETs, was determined immobilizing a mix of histones to the plate. Wells were coated overnight at 4 °C with 1 μg/µL of histones in PBS. The plates were washed with PBST. To block additional protein-binding sites, the wells were treated for 1 h with 2% BSA in PBS. The plates were then incubated for 1 h with 1 μg/well of each sample. Plates were washed three times with PBST, and the primary antibody (ab9400) was incubated for 1 h diluted 1:500. After three additional washes with PBST, the secondary antibody (ab6728) was incubated for 45 min diluted 1:1000. After washing, o-phenylenediamine dihydrochloride was added, and the absorbance at 490 nm was determined using an ELISA plate reader. To quantify NETs, Citrullinated Histone H3 (CitH3) (Clone 11D3) was measured by ELISA Kit (Cayman) following the manual instruction in supernatants of neutrophils induced towards NETosis adding or not PMA (see “Neutrophil’s isolation and NETosis” paragraph above). 

### 2.13. Interaction of AAT and Histones

After 1 h incubation with 1 μg/well of each sample, wells were washed, and an anti-AAT antibody conjugated with HRP (Abcam) was added. The wells were washed again, and, upon addition of the substrate, the complex read at 655 nm. The development of color in all wells containing the COVID-19 samples demonstrated the formation of the complex between histones and AAT. 

### 2.14. Confocal Microscopy

2 × 10^6^ neutrophils were activated with 100 nM PMA with or without 500 µg/mL fluorescent AAT for 4 h at 37 °C. Afterwards, neutrophils were washed with PBS and fixed with 4% of paraformaldehyde for 10 min. After three washes with PBS, cells were treated with blocking solution (1% BSA in PBS) for 1 h at room temperature. Cells were then stained with anti-HNE mAb (dilution 1:50—MA1-40220—Invitrogen) in 0.5% BSA in PBS for 1 h at room temperature. After three washes in PBS, secondary mAbs (anti-rabbit IgG H&L, Alexa Fluor^®^ 647—Invitrogen) were added in 0.5% BSA in PBS for 1 h at room temperature. Coverslips were then mounted using ProLong™ Gold Antifade Mountant with DAPI (Invitrogen) and analysed with confocal microscopy (Fluoview FV10i, Olympus, Tokyo, Japan).

## 3. Results

### 3.1. Analysis of HNE/AAT Balance in BALf of COVID-19 Patients

As a confirmation of previously reported data [4], the number of neutrophils in BALf of patients was found to be increased compared to (historical) controls [12]. The ELISA assay confirmed that free HNE was present in all 33 samples under investigation (see Table 2). In addition, the use of nanodrop technology allowed us to demonstrate that HNE was active in all specimens included in this study in the range of 0.022 to 3.69 mU/mg. The total protein amount, the amount of HNE, its specific activity, and the amount of NETs determined in the samples investigated are summarized in Table 2.

**Figure 1:**
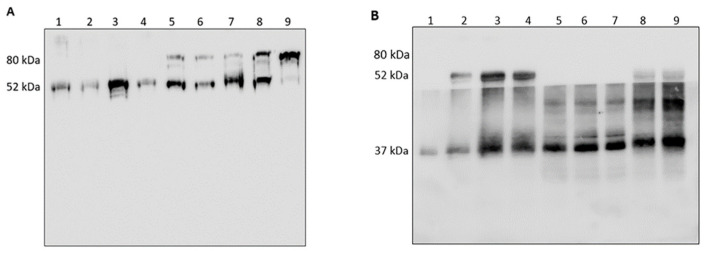
Panel (**A**). Western blotting with anti-AAT antibody. Lanes 1–4: COVID-19 samples not showing the complex (80 kDa band) upon addition of exogenous HNE. Lanes 5–8: BOS samples showing the complex (80 kDa band) upon addition of exogenous HNE. Lane 9: complex generated “in vitro” upon incubation of the HNE and AAT standard proteins. Panel (**B**). Western blotting with anti-AAT and anti-HNE antibodies. Lane 1: free HNE standard protein. Lanes 2–9: different COVID-19 samples showing an intensity increase of the HNE band upon incubation with scalar amounts of exogenous HNE.

Given the relevance of AAT in inhibiting free HNE [12], it seemed intriguing to explore to which extent AAT was able to inhibit HNE activity in these specimens. This was done on two COVID-19 samples (i.e., COVID-19 and COVID-24, chosen randomly among all available and representative of all others) through a gel electrophoresis run followed by Western blot analysis with the anti-AAT antibody. The result (lanes 1 and 3 of Figure 1, Panel A, for COVID-19 and COVID-24, respectively) showed the presence of a single band at approximately 52 kDa. The band, excised from the gel, digested with trypsin, and submitted to LC-MS as indicated in the MM section, was unambiguously identified as AAT (Table S2, see Supplementary Materials) thus confirming the results previously published [12]. Interestingly this profile remained unchanged by addition to these samples of exogenous HNE (2 µL; 0.2 mg/mL), the same band being evident in lanes 2 and 4 of the same panel. Thus, the apparent inability of these COVID-19 samples to form the HNE–AAT complex, in contrast to what previously reported [12], prompted a parallel loading of two COVID-19 BALf samples (representative of those available in the lab) and two samples from patients affected by a different lung disorder (BOS). As evidenced in lanes 5 and 7 of the same panel, BOS samples clearly showed an additional band at around 80 kDa. This band underwent the same treatment as indicated above and was identified as the HNE–AAT complex (see Table S1 of Supplementary Materials). Furthermore, the decrease in intensity of the 52 kDa band in favor of that at 80 kDa (lanes 6 and 8 of Figure 1) upon incubation with exogenous HNE (2 µL; 0.2 mg/mL) demonstrated the capacity of AAT present in these samples to bind HNE, in contrast to what observed in COVID-19 samples. As a control, the complex was generated in vitro upon incubation of the two (HNE and AAT) standard proteins in a stoichiometric ratio (lane 9 of Figure 1).

To validate the result and confirm the inability of AAT to complex free HNE in COVID-19 samples, three of the above specimens (COVID-19, COVID-21 and COVID-25, chosen randomly among all available) were incubated with scalar amounts of exogenous HNE (0.2 mg/mL), submitted to gel electrophoresis and western blot analysis with the anti-HNE and anti-AAT antibodies. As shown in panel B of Figure 1, the addition of exogenous HNE did not result in the formation of complex (80 kDa band) in any of the COVID-19 treated samples. The only observation was the increase in intensity of the HNE band. Analogous results were obtained with all other COVID-19 BALf specimens (data not shown).

### 3.2. Analysis of HNE and Identification of Complexes with Other Proteins 

**Figure 2:**
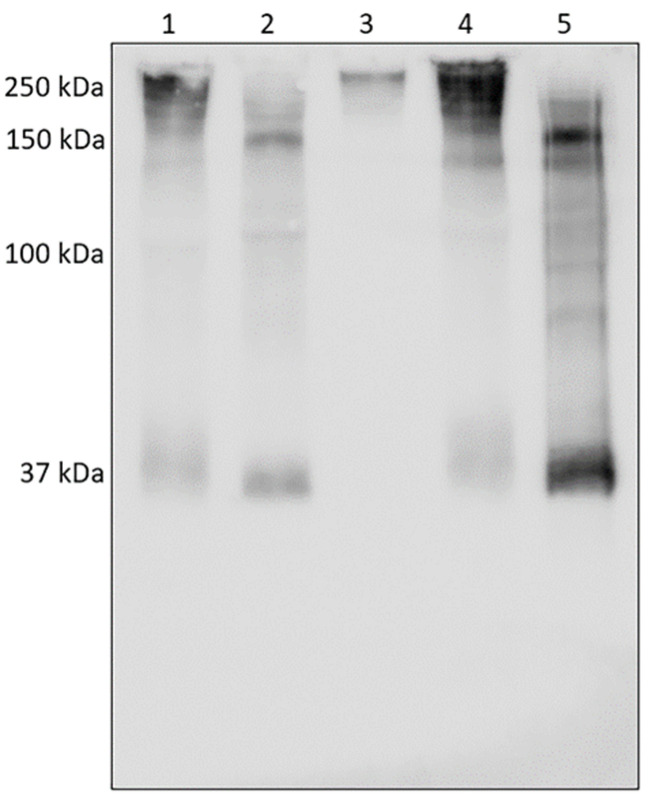
Western blotting with anti-HNE antibody. Five different COVID-19 samples show the presence of proteins in the range between 130 and 280 kDa which complexed with HNE.

The results seen above made us assume that HNE did not react with AAT in COVID-19 samples; thus, we firstly analyzed if HNE was bound to other BALf proteins. The fate of HNE in COVID-19 samples was investigated by running five COVID specimens (COVID-21, -22, -23, -24, -25, chosen at random among all available) on a gel that was blotted on a PVDF membrane and incubated with the anti-HNE antibody. This experiment (see the profiles of Figure 2), while confirming that the 80 kDa HNE–AAT complex was not present, additionally showed that most of the HNE in all specimens analyzed (except for one of them, see lane 5) was linked to other proteins forming aggregates whose molecular weight ranged between 130 and 280 kDa.

**Figure 3:**
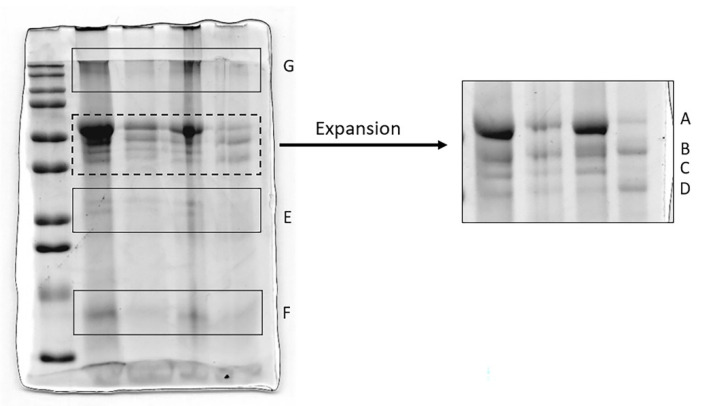
12.5% SDS-PAGE (on the left) showing the protein profile of the BALf samples considered. The gel on the right shows an expansion (obtained by running the samples on an 8% gel) of the region marked with a dotted line in the gel on the left. The bands excised, digested with Trypsin, and submitted to protein identification by LC-MS are those indicated by the letters A to G in both gels.

To gain insight into the nature of the non-AAT proteins that complexed HNE, these bands were excised from the gel, digested with trypsin and submitted to LC-MS for identification. Moreover, since the closeness of a few bands in the 12.5% gel (Figure 3, left) could prevent their unambiguous identification, this region was expanded, running a new gel with higher porosity (8%, right Figure). A total of seven bands (A to G, as indicated in the two Figures) were excised from the two gels and digested with trypsin following the procedure indicated in the MM section. The most significant proteins identified are shown in Table 3. The complete list of proteins identified is reported in Table S2 of Supplementary Materials. It can be observed that, in addition to α1-antitrypsin fragments (bands A, B, C, D, E, G), several other proteins, mostly acute phase proteins, were forming complexes with HNE. 

### 3.3. Analysis of AAT

The lack of the AAT–HNE complex in COVID samples led us to hypothesize that AAT could either be reacting with alternative targets or be inactivated by oxidation. Since one of the possible anti-inflammatory activities of AAT consists in the modulation of NET formation and activity, we focused our attention on the possible binding of AAT with the NET-derived proteins, in particular histones. Since AAT interaction with NET-derived proteins is not founded on covalent binding we did not expect to detect any band related to the histone–AAT complex in gel electrophoresis. Thus, to rule out the ability of AAT from COVID-19 patients to bind histones, an in vitro study was performed in which histones were immobilized on a 96-well plate and incubated with all BALf samples from COVID-19 patients.

**Figure 4:**
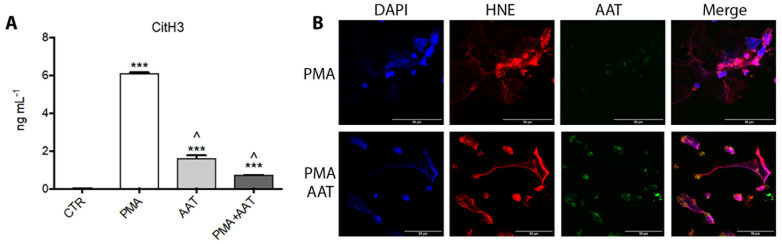
Panel (**A**). Quantification of CitH3 in neutrophils activated towards NETosis using PMA (100 nM) with or without AAT (500 µg/mL) by ELISA assay after 4 h of incubation. Data are represented as mean of three independent replicates ± standard deviation. *** *p* < 0.01 vs. CTR; ^ *p* < 0.01 vs. PMA. Panel (**B**). Confocal microscopy images of neutrophils activated towards NETosis with PMA adding fluorescent AAT (green). DNA was labeled with DAPI and HNE with specific antibody (red). Scale bar = 50 µm.

AAT–NET interaction was finally confirmed in vitro by NET release assay. Neutrophils were further incubated with PMA to induce NETosis in the presence or absence of AAT. By means of citrullinated histone H3 quantification with an ELISA assay, we observed that the presence of AAT significantly reduced the number of histones released by neutrophils (Figure 4A), while at confocal microscopy analysis no significant reduction of NET release was detectable. We, therefore, analyzed NET formation in the presence of fluorescent AAT and documented the ability of this protein to link to NETs but not to decrease their formation rate (Figure 4B). These results suggest the possibility that AAT binding to NET proteins (including histones) might somehow mask the Ab-binding epitope in the ELISA assay (Figure 4A).

## 4. Discussion

COVID-19 is known to have a powerful inflammatory response, and an important role has been attributed to neutrophils and their mediators, like HNE [13,14,15]. As we previously assessed, AAT can interact with neutrophil elastase in BALf from lung transplant recipients who developed BOS [12]. In the current study we initially aimed to test the balance between AAT/HNE in severe COVID-19. BALf from patients who tested positive for SARS-CoV2 and admitted to ICU for acute respiratory distress syndrome (ARDS) were analyzed. Interestingly, contrary to what was expected and what was described in other clinical and experimental settings including other forms of ARDS [47,48], we observed that in severe SARS-CoV2 infection the AAT-HNE complex is lacking both in untreated samples and after sample pre-treatment with HNE. This might suggest that AAT in BALf of COVID-19 patients was not active. As expected, free HNE was present in these samples and endowed with full proteolytic activity in all specimens analyzed, in accordance with previous studies [14,15]. HNE is known to be harmful to lung tissues [9,10,11,12] and, based on our results, we can speculate that patients affected by severe COVID-19 are exposed to uncontrolled proteolytic lung damage due to the prevention of the formation of a protective complex with AAT. Our results are in line with previous evidence showing a significant increase in neutrophil elastase levels in BALf from acute respiratory distress syndrome (ARDS) caused by other noxae pathogenae [49]. Similarly, in this study, conserved neutrophil elastase activity has been detected despite the presence of elevated amounts of α1-antitrypsin, and this paradox was attributed to the binding of HNE to α2-macroglobulin, which typically does not have protease activity against small-molecular-mass substrates. 

However, other possible explanations for the lack of the 80 kDa band after incubation of BALf samples with exogenous HNE were explored, and we hypothesized that AAT could be inactivated or involved in binding with other macromolecules. The first possibility has been ruled out by demonstrating that AAT can bind to NETs, specifically to histones associated with NETs. Interestingly, our results showed the presence of a non-covalent binding with histones. It is known that citrullinated histones, a fundamental component of NETs, can cause injury to distant sites when released into the blood [50]. Whether the binding of AAT to NET-histones might at least in part prevent their damage has to be assessed in specifically designed studies. In fact, the exact biologic role of the binding of AAT to NETs is still unclear and we have demonstrated, in accordance with previous observations [34], that AAT does not prevent NET formation. Data on the protective activity of AAT towards NET-induced injury are still inconclusive. Nevertheless, we can at least hypothesize that, because of the binding with histone, a steric hindrance of AAT with consequent hiding of the HNE specific binding site might prevent the formation of complexes. 

Another possible explanation of the inability of AAT to form complexes with elastase is its inactivation by oxidation of the methionine 351 or methionine 358 residues. This pattern of inactivation has been described in vitro [51] and can be hypothesized in the context of severe neutrophil activation and high release of reactive oxygen species such as in the case of COVID-19. However, clear evidence of AAT oxidation in the alveolar microenvironment of COVID-19 is still lacking. This hypothesis is supported by the work of Cochrane et al. [47] that indirectly described the presence of oxidized AAT in BALf from ARDS patients by restoring the protease activity after treatment with methionyl sulfoxide-peptide reductase. Due to the limited amount of protein that could be purified by COVID-19 BALf samples, we were not able to explore this issue by means of the available techniques (i.e., LC-MS), and, to address this mechanism of inactivation in the specific context of COVID-19 patients, further studies will be planned. In accordance with other studies [14,15], an increased level of HNE (that is known to be harmful to lung tissues) was found in COVID-19 BALf samples [9,10,11,12]. However, based on our findings, rather than being bound to AAT, the vast amount of HNE binds a good number of other proteins among which lactoferrin, hemopexin, haptoglobin, and human polymeric immunoglobulin receptor appear to be the most relevant. Given the role of these acute phase proteins in the regulation of the innate inflammatory reaction, we could speculate a potential pathogenic role of HNE in the modulation of their activity, possibly limiting their availability. This could be the case of human lactoferrin (LF), a protein that, due to its capacity to act as antibacterial, antiviral, antifungal, anti-inflammatory, and anti-carcinogenic, plays an important role in host defense [52]. It is also associated to NETs and, given its ability to trap iron, it creates iron-deficient areas that reduce bacterial growth. On the other hand, LF also seems to suppress NET release by forming a shell around activated neutrophils. All together, these properties make lactoferrin an intrinsic inhibitor of NET release into circulation and may, therefore, be central in their control [53]. In addition, LF shows a strong antiviral activity against a broad spectrum of DNA and RNA viruses by inhibiting the entry of viral particles into host cells, either by direct attachment to the viral particles or by blocking their cellular receptors [54]. These viruses typically use common molecules on the cell membrane to facilitate their invasion into cells, including heparan sulfate proteoglycans HSPGs. It has been shown that LF is able to prevent the internalization of some viruses by binding to (HSPGs) [55]. Given that HSPGs also play an important role in the process of SARS-CoV-2 host cell entry [54,55], LF might play an important role in the defense against SARS-CoV-2 by not only sequestering iron and inflammatory molecules that are severely increased during the cytokine burst but also blocking the entry of viruses in the host cells by preventing its initial attachment and accumulation on the cell membrane. It seems logical to hypothesize that, if this protein is sequestered in whole or in part by HNE, there could be less of it available to defend the organism from the attack of the virus. Hemopexin (HPX) is a protein whose synthesis is induced after an inflammatory event [56] and that has a high affinity for the heme group that, among other features, amplifies the innate immune responses induced by toll-like receptor 4 causing adverse immune reactions such as the migration of leukocytes and the production of adhesion molecules and cytokines [57]. In the pulmonary system, this results in increased vascular permeability, interstitial edema, and migration of inflammatory cells [58]. Conditions that can result in the liberation of free heme include sepsis and endotoxemia. It has been reported that patients affected by COVID-19 pneumonia may develop endotoxemia that, in turn, can cause severe sepsis. As a result of endotoxemia, these patients undergo the so-called “cytokine storm” by releasing high levels of proinflammatory cytokines, causing multiple organ failures [59]. If available in appropriate concentration, HPX binds to the heme group and blocks the production of proinflammatory cytokines, thus reducing the pro-inflammatory response to heme accumulation and playing a protective role in chronic airway disease. Haptoglobin (Hp) is another protein that belongs to the acute-phase protein family whose levels may be decreased in patients with COVID-19, due to its complex with HNE. It seems that Hp also suppresses macrophage functions such as lipopolysaccharides-induced production of tumor necrosis factor alpha (TNF-α), proliferation, cytokine production by T cells, and proliferation of B cells [60,61,62]. Of particular interest was the finding of human polymeric immunoglobulin receptor (pIgR), a type I transmembrane protein whose major function is to bind polymeric Ig (pIg) to facilitate their transport across the epithelium [63]. According to some authors, HNE and other proteinases secreted by granulocytes cleave pIgR-pig, promoting the release of polymeric IgA and IgM from their receptor [64,65]. Fragments of IgA and IgM have been identified by LC-MS. Fibrinogen alpha chain (FGA protein), one of the components of fibrinogen, a “positive” acute-phase protein, whose blood levels rise in response to systemic inflammation and tissue injury was also identified under a high-molecular weight band. The presence of HNE associated with this protein comes as no surprise. In fact, it has been demonstrated that fibrinogen and coagulation factors of the intrinsic pathway associate with the DNA, histones or proteases comprising NETs. In fact, in addition to their microbial clearance functions, NETs have been shown to promote thrombin generation, fibrin formation, and platelet activation in both in vitro and in vivo models to promote thrombus formation [66]. The presence of high levels of NETs causing sputum thickening, pulmonary inflammation, and hindrance to gaseous exchange was also shown in a recent quantitative proteomic analysis of the sputum from patients with severe COVID-19 [63]. Interestingly, the treatment of patients with recombinant human DNase reduced NETs and was associated with improved recovery and inflammation, thus suggesting a possible therapeutic implication in COVID-19 disease [67].

In conclusion, based on our data we can hypothesize that a supplemental therapy with exogenous AAT, that has been suggested in COVID-19 patients, might be unsuccessful due to the following reasons: AAT might be inactivated by the highly inflammatory microenvironment either by binding to NETs or by oxidation and, on the other side, HNE might be inaccessible due to interaction with other proteins. Further studies are in progress to unravel these issues. 

## Figures and Tables

**Table 1:** Demographic and clinical features (including age, gender, % of macrophages, lymphocytes, neutrophils, eosinophils, days after incubation and P/F) of patients considered in this study.

Numbering	Age	Gender	Macrophages (%)	Lymphocytes (%)	Neutrophils (%)	Eosinophils (%)	Days after Intubation	P/F **
COVID-01	67	M	n.d.	n.d.	n.d.	n.d.	1	123
COVID-02	54	M	9	7	81	2	2	107
COVID-03	41	M	41	0	60	0	3	92
COVID-04	69	M	n.d.	n.d.	n.d.	n.d.	1	107
COVID-05	60	M	15	0	85	0	4	68
COVID-06	50	F	2	6	92	0	3	180
COVID-07	47	F	23	12	65	0	3	98
COVID-08	66	M	n.d.	n.d.	n.d.	n.d.	2	111
COVID-09	78	F	33	24	43	0	2	209
COVID-10	51	F	34	8	58	0	3	142
COVID-11	60	M	64	25	11	0	3	123
COVID-12	66	F	45	29	26	0	4	71
COVID-13	60	M	81	9	10	0	2	73
COVID-14	53	M	56	13	31	0	2	200
COVID-15	43	M	17	10	74	0	3	113
COVID-16	60	M	n.d.	n.d.	n.d.	n.d.	1	145
COVID-17	61	M	33	12	61	0	1	192
COVID-18	72	F	10	14	77	0	1	300
COVID-19	56	F	n.d.	n.d.	n.d.	n.d.	1	132
COVID-20	60	M	18	13	81	0	2	145
COVID-21	28	M	n.d.	n.d.	n.d.	n.d.	1	188
COVID-22	65	M	14	10	76	0	3	121
COVID-23	55	M	68	10	22	0	1	185
COVID-24	63	M	35	14	51	0	1	176
COVID-25	80	M	8	1	91	0	2	148
COVID-26	64	M	8	2	90	0	2	200
COVID-27	67	M	n.d.	n.d.	n.d.	n.d.	2	179
COVID-28	29	M	18	4	78	0	1	210
COVID-29	74	M	19	6	75	0	4	300
COVID-30	70	M	13	5	82	0	3	192
COVID-31	63	M	n.d.	n.d.	n.d.	n.d.	2	175
COVID-32	69	M	n.d.	n.d.	n.d.	n.d.	2	187
COVID-33	68	F	12	11	77	0	1	158

n.d. Not determined; ** pO_2_/FiO_2_.

**Table 2:** Total protein concentration; HNE concentration and specific activity; NETs concentration for each sample considered in this study.

Numbering	BCA (mg/mL)	Elastase (ng/mL)	Elastase Specific Activity (mU/mg)	NETs (Cit H3 ng/mL)
COVID-01	1.24	40.70	2.79	2.98
COVID-02	0.72	60.69	1.22	0.96
COVID-03	0.95	68.36	1.07	n.d.
COVID-04	2.64	7.92	1.34	n.d.
COVID-05	1.44	92.92	1.66	1.52
COVID-06	3.16	32.99	3.69	n.d.
COVID-07	0.39	29.44	1.10	0.32
COVID-08	0.52	11.82	0.04	0.13
COVID-09	1.29	82.29	0.74	0.05
COVID-10	1.67	29.19	0.41	0.10
COVID-11	0.38	2.65	3.68	0.06
COVID-12	0.36	6.63	0.64	0.06
COVID-13	0.38	2.99	0.32	n.d.
COVID-14	0.42	22.64	0.29	0.17
COVID-15	0.41	25.81	0.19	0.29
COVID-16	1.55	80.01	0.27	2.64
COVID-17	0.81	36.94	0.81	0.18
COVID-18	1.02	47.54	0.43	0.09
COVID-19	1.03	12.80	1.45	0.09
COVID-20	0.33	2.41	1.24	0.27
COVID-21	1.25	37.63	1.97	2.89
COVID-22	0.78	2.62	0.17	0.38
COVID-23	0.43	2.79	0.56	n.d.
COVID-24	1.69	13.50	0.80	1.06
COVID-25	0.99	13.60	2.04	0.16
COVID-26	0.54	2.02	1.37	n.d.
COVID-27	0.97	12.52	1.77	0.17
COVID-28	0.36	4.15	0.97	0.13
COVID-29	0.58	15.24	1.53	3.26
COVID-30	0.57	14.23	0.91	0.07
COVID-31	0.57	1.88	0.74	0.71
COVID-32	0.73	10.78	0.46	0.54
COVID-33	0.55	8.51	1.62	0.33

Values reported are the mean of three independent determinations. Standard deviation was within 5%. n.d. = not detectable.

**Table 3:** Most significant proteins identified by LC-MS/MS under the bands excised from the gels.

Accession Number	Gene Name	Protein Name	Score (%)	Coverage (%)	Peptide Count
tr|Q19KS2|Q19KS2_HUMAN	LTF	Lactoferrin	92	3.68	2
sp|P61626|LYSC_HUMAN	LYZ	Lysozyme C	99	23.65	5
sp|P02790|HEMO_HUMAN	HPX	Hemopexin	99	8.44	4
tr|Q8WW76|Q8WW76_HUMAN	FGA	Fibrinogen alpha chain	99	20.18	6
sp|P01833|PIGR_HUMAN	PIGR	Polymeric immunoglobulin receptor	99	4.32	4
tr|Q6NSB4|Q6NSB4_HUMAN	HP	haptoglobin (HP protein)	91	16.01	3
sp|P01024|CO3_HUMAN	C3	Complement C3	99	5.17	10
tr|H9KV48|H9KV48_HUMAN	SERPING1	Plasma protease C1 inhibitor	90	5.18	4
sp|P62807|H2B1C_HUMAN	H2BC4	Histone H2B	99	35.71	7
tr|B2R5B3|B2R5B3_HUMAN	H2AFB1	Histone H2A	99	16.92	4
sp|P62805|H4_HUMAN	H4C1	Histone H4	99	45.63	7
sp|P01009|A1AT_HUMAN	SERPINA1	Alpha-1-antitrypsin	99	22.73	13
sp|P08246|ELNE_HUMAN	ELANE	Human Neutrophil Elastase	69	12.36	4

## Data Availability

Not applicable.

## Supplementary Materials

The following supporting information can be downloaded at: https://www.mdpi.com/article/10.3390/cimb44050143/s1, Table S1: Identification of HNE and AAT by LC-MS; Table S2: List of all proteins identified in gel bands analyzed by LC-MS.

## Abbreviations

Bronchoalveolar lavage	BAL
Human neutrophil elastase	HNE
Alpha_1_-antitrypsin	AAT
Reactive oxygen species	ROS
Neutrophil extracellular traps	NETs
Bronchiolitis obliterans syndrome	BOS
Acute respiratory distress syndrome	ARDS
Human lactoferrin	LF
Hemopexin	HPX
Haptoglobin	Hp
Fibrinogen alpha chain	FGA protein
Phorbol myristate acetale	PMA
Trichloroacetic acid	TCA
Sodium dodecyl sulphate	SDS
Bromophenol blue	BPB
Acetonitrile	ACN
Formic acid	FA
