# Supplementary material for: Investigating the Link between Alpha-1 Antitrypsin and Human Neutrophil Elastase in Bronchoalveolar Lavage Fluid of COVID-19 Patients
Source: Curr Issues Mol Biol. 2022 May 10;44(5):2122–38. doi: 10.3390/cimb44050143 (PMC9164061; doi:10.3390/cimb44050143)
Supplement: Supplementary file 1 [file cimb-44-00143-s001.zip › cimb-1711117-supplementary.pdf]

Table S1. Identification of HNE and AAT by LC-MS.

Accession	Mass	Score (%)	Coverage (%)	Description	m/z	z	Peptide
sp|P01009|A1AT_HUMAN	46.737	99	14.83%	Alpha-1-antitrypsin OS=Homo sapiens			
	1345.9855				674	2	RLGMFNIQHCK
	920.99274				922	1	FLENEDR
	1777.9855				890	2	TDTSHHDQDHPTFNK
	1331.9783				445	3	LVDKFLEDVKK
	1077.9855				540	2	FLENEDRR
	1544.9783				516	3	SPLFMGKVVNPTQK
	851.9855				427	2	SASLHLPK
	1317.9855				660	2	LGMFNIQHCKK
sp|P08246|ELNE_HUMAN	28.518	62	4.87%	Neutrophil elastase OS=Homo sapiens			
	1545.9855				774	2	AVRVVLGAHNLSRR
	1389.9855				696	2	AVRVVLGAHNLSR

Table S2. List of all proteins identified in gel bands analyzed by LC-MS.

Bands	Molecular Weight	Accession	Mass (kDa)	Score (%)	Coverage (%)	Description
**A**	52 kDa	tr|H0YA55|H0YA55_HUMAN	51.461	99	38.11	Serum albumin (Fragment) OS=Homo sapiens OX=9606 GN=ALB PE=1 SV=1
		tr|F6KPG5|F6KPG5_HUMAN	66.531	99	37.44	Albumin (Fragment) OS=Homo sapiens OX=9606 PE=2 SV=1
		tr|Q13747|Q13747_HUMAN	22.828	99	31.98	Alpha-1 antitrypsin (Fragment) OS=Homo sapiens OX=9606 PE=2 SV=1
		sp|P01009|A1AT_HUMAN	46.737	99	22.73	Alpha-1-antitrypsin OS=Homo sapiens OX=9606 GN=SERPINA1 PE=1 SV=3
		sp|P01011|AACT_HUMAN	47.651	99	24.59	Alpha-1-antichymotrypsin OS=Homo sapiens OX=9606 GN=SERPINA3 PE=1 SV=2
		tr|B4DI57|B4DI57_HUMAN	63.437	99	19.09	cDNA FLJ54111, highly similar to Serotransferrin OS=Homo sapiens OX=9606 PE=2 SV=1
		sp|P02790|HEMO_HUMAN	51.676	99	8.44	Hemopexin OS=Homo sapiens OX=9606 GN=HPX PE=1 SV=2
		tr|S6AWF0|S6AWF0_HUMAN	25.491	94	6.28	IgG H chain OS=Homo sapiens OX=9606 PE=2 SV=1
		tr|Q5EFE5|Q5EFE5_HUMAN	52.363	92	6.95	Anti-RhD monoclonal T125 gamma1 heavy chain OS=Homo sapiens OX=9606 PE=2 SV=1
**B**	47 kDa	tr|H0YA55|H0YA55_HUMAN	51.461	99	35.9	Serum albumin (Fragment) OS=Homo sapiens OX=9606 GN=ALB PE=1 SV=1
		tr|F6KPG5|F6KPG5_HUMAN	66.531	99	33.68	Albumin (Fragment) OS=Homo sapiens OX=9606 PE=2 SV=1
		sp|P01009|A1AT_HUMAN	46.737	99	12.44	Alpha-1-antitrypsin OS=Homo sapiens OX=9606 GN=SERPINA1 PE=1 SV=3
		tr|B4DHZ6|B4DHZ6_HUMAN	47.388	99	9.2	Transferrin, isoform CRA_c OS=Homo sapiens OX=9606 GN=TF PE=2 SV=1
		sp|P02774|VTDB_HUMAN	52.918	99	3.8	Vitamin D-binding protein OS=Homo sapiens OX=9606 GN=GC PE=1 SV=2
		tr|Q6PYX1|Q6PYX1_HUMAN	38.162	99	17.24	Hepatitis B virus receptor binding protein (Fragment) OS=Homo sapiens OX=9606 PE=1 SV=1
		sp|P01011|AACT_HUMAN	47.651	99	5.67	Alpha-1-antichymotrypsin OS=Homo sapiens OX=9606 GN=SERPINA3 PE=1 SV=2
		tr|B7Z8Q4|B7Z8Q4_HUMAN	15.718	90	7.04	cDNA FLJ56652, highly similar to Hemopexin OS=Homo sapiens OX=9606 PE=2 SV=1
		tr|B4E1V0|B4E1V0_HUMAN	33.025	89	2.67	cDNA FLJ54839, highly similar to Lactotransferrin OS=Homo sapiens OX=9606 PE=2 SV=1
**C**	42 kDa	tr|H0YA55|H0YA55_HUMAN	51.461	99	31.72	Serum albumin (Fragment) OS=Homo sapiens OX=9606 GN=ALB PE=1 SV=1
		tr|Q56G89|Q56G89_HUMAN	69.084	99	30.54	Serum albumin OS=Homo sapiens OX=9606 PE=2 SV=1
		sp|P01009|A1AT_HUMAN	46.737	99	8.85	Alpha-1-antitrypsin OS=Homo sapiens OX=9606 GN=SERPINA1 PE=1 SV=3
		tr|G3V3A0|G3V3A0_HUMAN	23.406	99	8.29	Alpha-1-antichymotrypsin OS=Homo sapiens OX=9606 GN=SERPINA3 PE=1 SV=1
		tr|B4E1V0|B4E1V0_HUMAN	33.025	90	2.67	cDNA FLJ54839, highly similar to Lactotransferrin OS=Homo sapiens OX=9606 PE=2 SV=1
		tr|A0A0A0MS07|A0A0A0MS07_HUMAN	31.983	75	2.37	Immunoglobulin heavy constant gamma 1 (Fragment) OS=Homo sapiens OX=9606 GN=IGHG1 PE=1 SV=1
**D**	37 kDa	tr|H0YA55|H0YA55_HUMAN	51.461	99	31.5	Serum albumin (Fragment) OS=Homo sapiens OX=9606 GN=ALB PE=1 SV=1
		tr|F6KPG5|F6KPG5_HUMAN	66.531	99	30.09	Albumin (Fragment) OS=Homo sapiens OX=9606 PE=2 SV=1
		sp|P01009|A1AT_HUMAN	46.737	99	10.77	Alpha-1-antitrypsin OS=Homo sapiens OX=9606 GN=SERPINA1 PE=1 SV=3
		sp|P01011|AACT_HUMAN	47.651	99	7.8	Alpha-1-antichymotrypsin OS=Homo sapiens OX=9606 GN=SERPINA3 PE=1 SV=2
		tr|Q6N095|Q6N095_HUMAN	52.36	99	5.89	Uncharacterized protein OS=Homo sapiens OX=9606 GN=DKFZp686K03196 PE=1 SV=1

		tr|B4E1V0|B4E1V0_HUMAN	33.025	90	2.67	cDNA FLJ54839, highly similar to Lactotransferrin OS=Homo sapiens OX=9606 PE=2 SV=1
**E**	25-30 kDa	tr|H0YA55|H0YA55_HUMAN	51.461	99	31.72	Serum albumin (Fragment) OS=Homo sapiens OX=9606 GN=ALB PE=1 SV=1
		tr|Q56G89|Q56G89_HUMAN	69.084	99	30.54	Serum albumin OS=Homo sapiens OX=9606 PE=2 SV=1
		sp|P01857|IGHG1_HUMAN	36.106	99	26.36	Immunoglobulin heavy constant gamma 1 OS=Homo sapiens OX=9606 GN=IGHG1 PE=1 SV=1
		tr|Q6PYX1|Q6PYX1_HUMAN	38.162	99	25.00	Hepatitis B virus receptor binding protein (Fragment) OS=Homo sapiens OX=9606 PE=1 SV=1
		sp|P01009|A1AT_HUMAN	46.737	99	15.55	Alpha-1-antitrypsin OS=Homo sapiens OX=9606 GN=SERPINA1 PE=1 SV=3
		tr|G3V3A0|G3V3A0_HUMAN	23.406	99	30.73	Alpha-1-antichymotrypsin OS=Homo sapiens OX=9606 GN=SERPINA3 PE=1 SV=1
		tr|A0A5J6KJ24|A0A5J6KJ24_HUMAN	38.388	99	16.52	IGHG3 (Fragment) OS=Homo sapiens OX=9606 PE=2 SV=1
		sp|P61626|LYSC_HUMAN	16.537	99	23.65	Lysozyme C OS=Homo sapiens OX=9606 GN=LYZ PE=1 SV=1
		sp|P01859|IGHG2_HUMAN	35.901	99	9.82	Immunoglobulin heavy constant gamma 2 OS=Homo sapiens OX=9606 GN=IGHG2 PE=1 SV=2
		sp|P01861|IGHG4_HUMAN	35.941	99	9.79	Immunoglobulin heavy constant gamma 4 OS=Homo sapiens OX=9606 GN=IGHG4 PE=1 SV=1
		tr|Q19KS2|Q19KS2_HUMAN	39.143	92	3.68	Lactoferrin (Fragment) OS=Homo sapiens OX=9606 PE=2 SV=1
		tr|Q5H9B4|Q5H9B4_HUMAN	10.607	90	11.58	Metalloproteinase inhibitor 1 (Fragment) OS=Homo sapiens OX=9606 GN=TIMP1 PE=1 SV=8
		sp|P08246|ELNE_HUMAN	28.518	19	12.36	Neutrophil elastase OS=Homo sapiens OX=9606 GN=ELANE PE=1 SV=1;
**F**	10 kDa	sp|P61626|LYSC_HUMAN	16.537	99	31.76	Lysozyme C OS=Homo sapiens OX=9606 GN=LYZ PE=1 SV=1
		sp|P68871|HBB_HUMAN	15.998	99	44.9	Hemoglobin subunit beta OS=Homo sapiens OX=9606 GN=HBB PE=1 SV=2
		sp|P62807|H2B1C_HUMAN	13.906	99	35.71	Histone H2B type 1-C/E/F/G/I OS=Homo sapiens OX=9606 GN=H2BC4 PE=1 SV=4
		tr|Q56G89|Q56G89_HUMAN	69.084	99	6.08	Serum albumin OS=Homo sapiens OX=9606 PE=2 SV=1
		sp|P62805|H4_HUMAN	11.367	99	45.63	Histone H4 OS=Homo sapiens OX=9606 GN=H4C1 PE=1 SV=2
		tr|A2KBC0|A2KBC0_HUMAN	25.205	99	9.66	Anti-(ED-B) scFV (Fragment) OS=Homo sapiens OX=9606 PE=2 SV=1
		tr|B2R5B3|B2R5B3_HUMAN	14.111	99	16.92	Histone H2A OS=Homo sapiens OX=9606 PE=2 SV=1
		tr|V9HW68|V9HW68_HUMAN	51.716	99	5.96	Epididymis luminal protein 214 OS=Homo sapiens OX=9606 GN=HEL-214 PE=1 SV=1
		tr|Q6GMX6|Q6GMX6_HUMAN	51.083	99	4.95	IGH@ protein OS=Homo sapiens OX=9606 GN=IGH@ PE=1 SV=1
		tr|A0A5C2G7S1|A0A5C2G7S1_HUMAN	11.676	98	23.36	IGL c60_light_IGKV3-15_IGKJ1 (Fragment) OS=Homo sapiens OX=9606 PE=2 SV=1
		tr|A0A5C2G572|A0A5C2G572_HUMAN	11.556	98	17.76	IGL c3015_light_IGKV3-20_IGKJ1 (Fragment) OS=Homo sapiens OX=9606 PE=2 SV=1
		tr|A0A5C2G8U7|A0A5C2G8U7_HUMAN	11.714	98	17.76	IGL c256_light_IGKV3-15_IGKJ1 (Fragment) OS=Homo sapiens OX=9606 PE=2 SV=1
		tr|A0A5C2GIX8|A0A5C2GIX8_HUMAN	13.391	94	8.8	IG c75_heavy_IGHV3-23_IGHD1-26_IGHJ3 (Fragment) OS=Homo sapiens OX=9606 PE=2 SV=1
		tr|A0A5C2G223|A0A5C2G223_HUMAN	11.823	93	23.15	IGL c383_light_IGKV3-20_IGKJ2 (Fragment) OS=Homo sapiens OX=9606 PE=2 SV=1
		sp|P01011|AACT_HUMAN	47.651	90	3.07	Alpha-1-antichymotrypsin OS=Homo sapiens OX=9606 GN=SERPINA3 PE=1 SV=2
		tr|A0A0C4DGL8|A0A0C4DGL8_HUMAN	38.452	90	4.03	Haptoglobin OS=Homo sapiens OX=9606 GN=HP PE=1 SV=1
		sp|P08246|ELNE_HUMAN	28.518	22	9.36	Neutrophil elastase OS=Homo sapiens OX=9606 GN=ELANE PE=1 SV=1;
**G**	150-250 kDa	tr|H0YA55|H0YA55_HUMAN	51.461	99	36.78	Serum albumin (Fragment) OS=Homo sapiens OX=9606 GN=ALB PE=1 SV=1

	tr|C9JKR2|C9JKR2_HUMAN	47.287	99	35.01	Albumin, isoform CRA_k OS=Homo sapiens OX=9606 GN=ALB PE=1 SV=1
	tr|B7WNR0|B7WNR0_HUMAN	56.212	99	34.41	Serum albumin OS=Homo sapiens OX=9606 GN=ALB PE=1 SV=1
	tr|F6KPG5|F6KPG5_HUMAN	66.531	99	34.36	Albumin (Fragment) OS=Homo sapiens OX=9606 PE=2 SV=1
	sp|P01857|IGHG1_HUMAN	36.106	99	28.79	Immunoglobulin heavy constant gamma 1 OS=Homo sapiens OX=9606 GN=IGHG1 PE=1 SV=1
	tr|B4DHZ6|B4DHZ6_HUMAN	47.388	99	28.77	Transferrin, isoform CRA_c OS=Homo sapiens OX=9606 GN=TF PE=2 SV=1
	tr|Q6PYX1|Q6PYX1_HUMAN	38.162	99	26.72	Hepatitis B virus receptor binding protein (Fragment) OS=Homo sapiens OX=9606 PE=1 SV=1
	tr|B4DI57|B4DI57_HUMAN	63.437	99	24.69	cDNA FLJ54111, highly similar to Serotransferrin OS=Homo sapiens OX=9606 PE=2 SV=1
	tr|B4E1B2|B4E1B2_HUMAN	74.832	99	24.04	cDNA FLJ53691, highly similar to Serotransferrin OS=Homo sapiens OX=9606 PE=2 SV=1
	tr|Q6MZV7|Q6MZV7_HUMAN	52.121	99	23.47	Uncharacterized protein DKFZp686C11235 OS=Homo sapiens OX=9606 GN=DKFZp686C11235 PE=1 SV=1
	sp|P01009|A1AT_HUMAN	46.737	99	16.51	Alpha-1-antitrypsin OS=Homo sapiens OX=9606 GN=SERPINA1 PE=1 SV=3
	sp|P01011|AACT_HUMAN	47.651	99	21.75	Alpha-1-antichymotrypsin OS=Homo sapiens OX=9606 GN=SERPINA3 PE=1 SV=2
	tr|S6BGF9|S6BGF9_HUMAN	22.743	99	25.35	IgG L chain OS=Homo sapiens OX=9606 PE=2 SV=1
	tr|S6BAP8|S6BAP8_HUMAN	22.852	99	24.88	IgG L chain OS=Homo sapiens OX=9606 PE=2 SV=1
	tr|W8QEY1|W8QEY1_HUMAN	78.382	99	19.13	Lactoferrin OS=Homo sapiens OX=9606 PE=2 SV=1
	tr|Q96K68|Q96K68_HUMAN	53.088	99	9.51	
	tr|Q6IPQ0|Q6IPQ0_HUMAN	24.824	99	30.51	IGL@ protein OS=Homo sapiens OX=9606 GN=IGL@ PE=2 SV=1
	sp|P61626|LYSC_HUMAN	16.537	99	31.08	Lysozyme C OS=Homo sapiens OX=9606 GN=LYZ PE=1 SV=1
	tr|A7Y9J9|A7Y9J9_HUMAN	648.809	99	1.26	Mucin 5AC, oligomeric mucus/gel-forming OS=Homo sapiens OX=9606 GN=MUC5AC PE=4 SV=1
	tr|Q6P5S8|Q6P5S8_HUMAN	25.773	99	25.85	IGK@ protein OS=Homo sapiens OX=9606 GN=IGK@ PE=1 SV=1
	tr|Q6MZU6|Q6MZU6_HUMAN	51.099	99	7.76	Uncharacterized protein DKFZp686C15213 OS=Homo sapiens OX=9606 GN=DKFZp686C15213 PE=2 SV=1
	sp|P02790|HEMO_HUMAN	51.676	99	8.01	Hemopexin OS=Homo sapiens OX=9606 GN=HPX PE=1 SV=2
	tr|A0A590UJZ9|A0A590UJZ9_HUMAN	166.577	99	2.82	Deleted in malignant brain tumors 1 protein OS=Homo sapiens OX=9606 GN=DMBT1 PE=1 SV=1
	sp|P01024|CO3_HUMAN	187.147	99	5.17	Complement C3 OS=Homo sapiens OX=9606 GN=C3 PE=1 SV=2
	tr|Q8WW76|Q8WW76_HUMAN	24.695	99	20.18	FGA protein OS=Homo sapiens OX=9606 GN=FGA PE=2 SV=1
	sp|P01833|PIGR_HUMAN	83.284	99	4.32	Polymeric immunoglobulin receptor OS=Homo sapiens OX=9606 GN=PIGR PE=1 SV=4
	tr|S6AWF0|S6AWF0_HUMAN	25.491	94	6.28	IgG H chain OS=Homo sapiens OX=9606 PE=2 SV=1
	sp|P98088|MUC5A_HUMAN	585.574	92	1.1	Mucin-5AC OS=Homo sapiens OX=9606 GN=MUC5AC PE=1 SV=4
	tr|Q6NSB4|Q6NSB4_HUMAN	31.382	91	16.01	HP protein OS=Homo sapiens OX=9606 GN=HP PE=2 SV=1
	tr|B7Z5Q2|B7Z5Q2_HUMAN	108.822	91	5.6	cDNA FLJ58075, highly similar to Ceruloplasmin OS=Homo sapiens OX=9606 PE=2 SV=1
	tr|B4E1D3|B4E1D3_HUMAN	54.253	90	6.96	cDNA FLJ53952, highly similar to Fibrinogen beta chain OS=Homo sapiens OX=9606 PE=2 SV=1
	tr|H9KV48|H9KV48_HUMAN	50.989	90	5.18	Plasma protease C1 inhibitor OS=Homo sapiens OX=9606 GN=SERPING1 PE=1 SV=1
	tr|A0A3G5AXY7|A0A3G5AXY7_HUMAN	8.228	83	33.78	Hemoglobin subunit beta (Fragment) OS=Homo sapiens OX=9606 GN=HBB PE=3 SV=1

	sp|P08246|ELNE_HUMAN	28.518	16	17.98	Neutrophil elastase OS=Homo sapiens OX=9606 GN=ELANE PE=1 SV=1;
